# A machine learning-driven early warning system for cryptocaryoniasis in marine aquaculture

**DOI:** 10.1186/s13071-025-07124-z

**Published:** 2025-11-26

**Authors:** Xiao Xie, Bo Zhang, Xingyu Wang, Yunyan Jiang, Kurt Buchmann, Suming Zhou, Yuezhuo Li, Fei Yin, Jorge Galindo-Villegas

**Affiliations:** 1 School of Marine Sciences, National Demonstration Center for Experimental (Aquaculture) Education, Ningbo University, 169 South Qixing Road, Ningbo, 315832 People’s Republic of China; 2 Laboratory of Aquatic Pathobiology, Department of Veterinary and Animal Sciences, Faculty of Health and Medical Sciences, University of Copenhagen, 1870 Frederiksberg C, Denmark; 3 Department of Genomics, Faculty of Biosciences and Aquaculture, Nord University, 8049 Bodø, Norway

**Keywords:** Aquaculture diseases, *Cryptocaryon irritans*, Disease prediction, Epidemiology, Machine learning models, Parasitology, Random forest, Sustainability

## Abstract

**Background:**

Disease outbreaks, particularly cryptocaryoniasis caused by the ciliate *Cryptocaryon irritans*, pose significant barriers to sustainable marine fish aquaculture, undermining productivity, profitability, and biosecurity. Despite its impact, early warning tools for parasitic diseases leveraging advanced technologies remain underdeveloped.

**Methods:**

We developed a machine learning (ML)-driven early warning system for cryptocaryoniasis, integrating seven years of outbreak surveillance data (*n* = 429 events from 2016 to 2023) with 17 high-resolution oceanographic predictors influencing parasite life cycles along China’s coast. Five supervised ML models: logistic regression (LR), support vector machine (SVM), random forest (RF), XGBoost (XGB), and artificial neural network (ANN), were trained using cross-validation and benchmarked in commercial open-sea cages and recirculating aquaculture systems (RAS).

**Results:**

The RF model achieved the highest sensitivity (98.6%), with RF and XGB excelling in F1 scores (0.93 and 0.938, respectively), identifying stocking density, water temperature, salinity, pH, and novel predictors such as silicate and nitrate as key risk factors. The predictive engine was deployed as an open-source web-based platform, delivering weekly, spatially resolved outbreak forecasts. Field validation across 12 open-sea cage events and weekly RAS monitoring confirmed high predictive accuracy (91.67% in sea cages; 87.5% in RAS), revealing seasonal and latitudinal disease trends.

**Conclusions:**

This study establishes a robust, scalable framework for real-time disease forecasting in marine aquaculture, adaptable to other aquatic pathogen-host species to support parasite surveillance and precision health management across diverse global aquaculture systems. While further validation with larger datasets and integration of pathogen and host data will enhance future models, this system provides a flexible foundation for advancing disease control in aquatic environments.

**Graphical Abstract:**

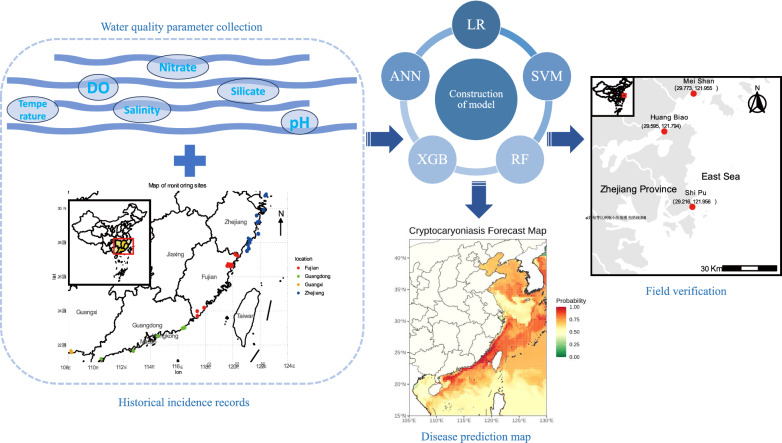

**Supplementary Information:**

The online version contains supplementary material available at 10.1186/s13071-025-07124-z.

## Background

Aquaculture of high-valued marine fish is expanding rapidly to meet global demand for sustainable animal protein [1]. However, disease outbreaks undermine productivity, resilience, and profitability, particularly in high-density coastal systems [2]. Among the most economically damaging is cryptocaryoniasis, caused by the ciliate parasite *Cryptocaryon irritans*. This parasite infects a broad range of teleost hosts and is widespread in tropical and temperate waters.

*Cryptocaryon irritans* is an obligate ectoparasite that invades the gills, skin, and fins, triggering necrosis, osmoregulatory dysfunction, and respiratory collapse [3–5]. Its rapid, temperature-sensitive life cycle, comprising trophont, protomont, tomont, and theront stages, facilitates explosive transmission dynamics to the point that a single tomont may release hundreds of infective theronts within days [6]. Once clinical symptoms emerge, tissue damage is often irreversible, and treatment options are limited, highlighting the urgent need for early detection and intervention tools.

Cryptocaryoniasis outbreaks exhibit temporal and spatial patterns driven by complex interactions among host susceptibility, pathogen biology, and environmental factors [7]. Epidemiological evidence suggests that outbreaks follow predictable seasonal patterns, typically between May and October, and are strongly modulated by abiotic variables including water temperature [8], salinity [9], oxygen, and pH [10]. However, the relationships among these factors are nonlinear, high-dimensional, and dynamic, conditions that challenge traditional statistical approaches to outbreak prediction.

Although *C. irritans* is not zoonotic, its management is integral to aquatic biosecurity and the One Health framework, which links environmental integrity, animal health, and food safety [11]. These approaches must be further mainstreamed in aquatic “blue” food systems to ensure that disease prevention and control are systematically embedded in aquaculture design and governance [12]. Doing so will help minimize distributional inequities, particularly in low-resource farming settings, and advance the goals of Blue Transformation. Effective surveillance of such parasitic diseases contributes to ecosystem stability and supports responsible aquaculture practices that reduce dependence on chemotherapeutics, thereby aligning with goals of antimicrobial stewardship and sustainable food production [13].

Machine learning (ML) offers a promising alternative for aquatic disease forecasting, as it can learn latent patterns from complex datasets and improve continuously through data assimilation [14]. While ML has revolutionized terrestrial agricultural health monitoring, its use in marine aquaculture remains nascent [15]. Existing disease surveillance systems typically lack real-time environmental integration, are restricted to recirculating aquaculture systems (RAS), and provide little support for field-scale decision-making [16].

To address this gap, we introduced a real-time ML-based early warning system for cryptocaryoniasis, developed using 6 years of outbreak data (*n* = 429 events) from coastal China and 17 oceanographic and meteorological predictors. We trained and benchmarked five supervised learning models (logistic regression (LR), support vector machine (SVM), random forest (RF), XGBoost (XGB), and artificial neural network (ANN)) and validated their performance in both open-sea cage and RAS environments. The best-performing model was deployed on a user-facing web platform that delivers weekly, spatially explicit risk forecasts. Beyond cryptocaryoniasis, this modular system is adaptable to other aquatic pathogens and host species, providing a scalable foundation for precision health management across marine aquaculture.

## Methods

### Data acquisition

**Fig. 1:**
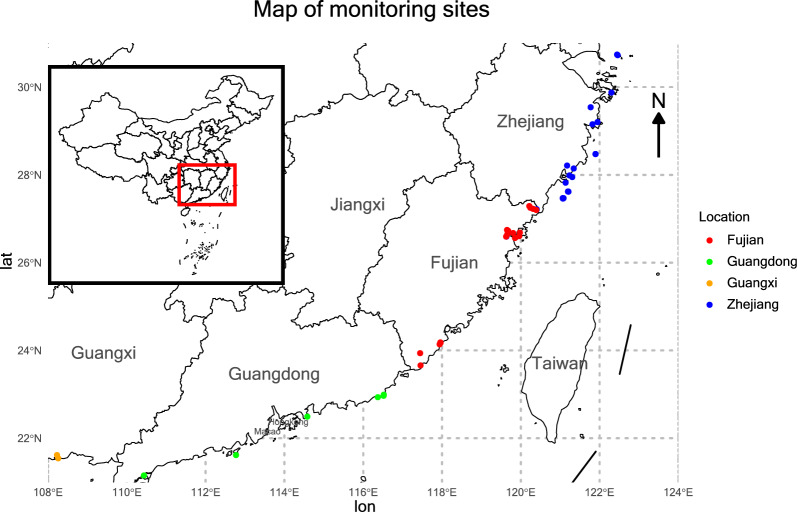
Geographic distribution of disease monitoring sites along the southeastern coast of China. Colored dots indicate sampling locations in four major aquaculture provinces: Zhejiang (*blue*), Fujian (*red*), Guangdong (*green*), and Guangxi (*orange*). The inset map shows the location of the study area within the national boundary of China. Owing to coastline generalization, some markers appear slightly inland; all sites represent marine aquaculture operations, with environmental variables extracted from the nearest Copernicus Marine grid cell (~5 km resolution)

The dataset, sourced from the National Fisheries Technology Extension Center, comprises 429 confirmed cryptocaryoniasis outbreak records in four different areas along China’s coast from 2016 to 2023 (Fig. 1). Outbreaks were confirmed via clinical signs and wet-mount microscopy by trained personnel, standardized across regions, following established protocols [4]. These records were collected from four major marine fish farming regions in China, namely Zhejiang, Fujian, Guangdong, and Guangxi provinces. These coastal zones represent high-density aquaculture hubs, where economically important fish species such as large yellow croaker (*Larimichthys crocea*) and groupers (*Epinephelinae* spp.) are intensively cultivated in net pens and sea cages. The epidemiological dataset includes four primary features: farm location, stocking density, culture method, and disease occurrence records. To enrich the dataset, we integrated environmental parameters from the Copernicus Marine Environment Monitoring Service, which provides global oceanographic and biogeochemical datasets [17]. Using the terra package in R [18, 19], we extracted site-specific environmental variables corresponding to the spatial coordinates and time of each outbreak record. The environmental variables retrieved included dissolved oxygen, pH, silicate, nitrate, and phosphate concentrations, net primary productivity, total chlorophyll, surface water temperature, seabed temperature, and salinity. In parallel, meteorological variables, such as precipitation, wind speed, relative humidity, and solar radiation, were extracted from the Copernicus Atmosphere Monitoring Services. In total, 17 predictor variables were assembled, encompassing both continuous and categorical data types. Outbreak status, determined by the presence or absence of cryptocaryoniasis, was treated as the target classification variable for model training and evaluation.

### Data preprocessing

Before the development of machine learning (ML) models, a robust data preprocessing pipeline was implemented to ensure both algorithmic efficacy and statistical validity. Given the heterogeneity in measurement scales across multivariate attributes, Min–Max normalization was applied to scale the data through linear transformation, thereby standardizing feature ranges without distorting relationships. Records with more than 70% missing values (12 out of 87) were excluded from the dataset. For the remaining data, variable-specific imputation approaches were applied. Environmental and meteorological variables were imputed using a random forest algorithm, which has been widely validated for handling nonlinear interactions and mixed-type data [20]. Aquaculture-related features with missing entries were imputed using variable means, following established statistical guidelines for continuous data with low proportions of missingness [21]. This strategy ensured that no record exceeded the 70% missingness threshold after filtering. Consequently, the proportion of imputed values per variable remained below this cutoff, thereby preserving dataset integrity while maximizing information retention. To address the inherent class imbalance between disease and nondisease instances, we employed the ROSE package in R [22], which generates synthetic samples of the minority class based on smoothed bootstrap resampling [23]. All data cleaning, manipulation, and transformation steps were performed using the dplyr [24] and tidyr [25] packages in R.

To minimize feature redundancy and model complexity, we applied recursive feature elimination (RFE) with a random forest classifier [26]. Feature importance was assessed via out-of-bag (OOB) error estimates, allowing for the iterative ranking and elimination of low-importance variables. This process yielded an optimal subset of predictive features, including stocking density, dissolved oxygen, chlorophyll, nitrate, phosphate, silicate, net biomass production, marine phytoplankton, water temperature, salinity, surface partial pressure of carbon dioxide, bottom water temperature, relative humidity, wind speed, solar radiation, and precipitation. The resulting dataset, preprocessed, normalized, and dimensionally reduced, was then imported and stored in MySQL to facilitate efficient access during ML model training and evaluation for downstream analyses.

### Model construction

To develop predictive models for cryptocaryoniasis outbreaks, we implemented five supervised ML algorithms using R-based libraries: logistic regression (LR), support vector machine (SVM) [27], random forest (RF) [28], extreme gradient boosting (XGBoost) [29], and artificial neural network (ANN) [30]. Among these, LR served as a baseline model for comparative benchmarking, providing a linear reference for evaluating the relative predictive performance of the more complex, nonlinear models. To ensure robust model generalization, a five-fold cross-validation strategy was employed. This approach enabled the optimization of hyperparameters through grid search, with model performance assessed on validation partitions using the presence or absence of cryptocaryoniasis as the binary target variable [31–33].

Given the inherent class imbalance in the dataset, model evaluation prioritized metrics that account for both error types, false positives (predicting and outbreak where none occurred) and false negatives (failing to detect a true outbreak). Accordingly, we reported accuracy, precision, recall (sensitivity), specificity, the F1-score, the area under the receiver operating characteristic curve (AUROC), and the area under the precision-recall curve (AUPRC), the latter being particularly informative in imbalanced classification contexts. Additional performance metrics, including accuracy, precision, sensitivity (recall), specificity, and the F1-score, are all derived from the confusion matrix. For interpretation: true positives (TP) represent correctly predicted disease occurrences, while false negatives (FN) represent missed outbreak events. Conversely, true negatives (TN) refer to accurately predicted disease absence, and false positives (FP) correspond to erroneous outbreak predictions [34, 35]. These indicators collectively inform the comparative evaluation of model discriminative power under field aquaculture disease surveillance conditions.

### Field sampling for model accuracy validation in open-sea cage and recirculating aquaculture system

**Fig. 2:**
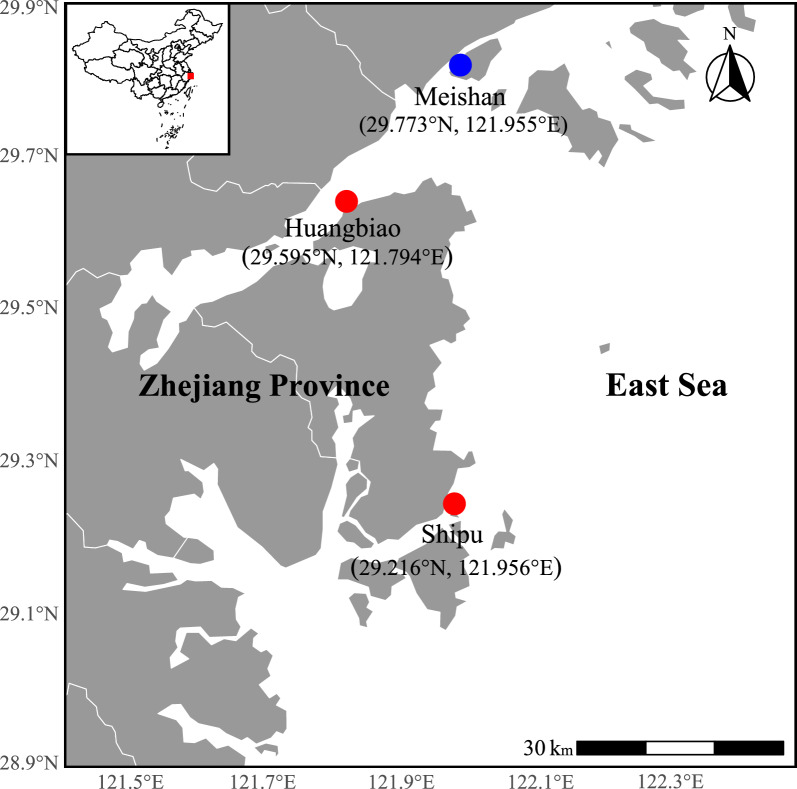
Field sampling sites used for model validation in different aquaculture systems. *Red dots* denote offshore cage farms cultivating large yellow croaker, while *blue dots* indicate the RAS pilot base at Ningbo University. Coordinates are shown in degrees (°) with cardinal notation (N/S, E/W). Land is outlined for clarity. Projection: WGS84 (Mercator)

To evaluate the real-world performance of the disease prediction model, field validation was conducted in two aquaculture settings: open-sea cage systems and RAS. For the open-sea validation, two commercial large yellow croaker farms located in Ningbo, Zhejiang, China, were selected (Fig. 2). Water sampling was conducted monthly from June to November 2022 (Table 1). At each site, five random sampling points were selected within the cage array, with data aggregated for analysis. Water was collected using a 2-m depth sub-surface Van Dorn water sampler, targeting the fish rearing zone. After registering the water temperature on-site, collected samples were immediately transported to the laboratory and analyzed for key hydrological parameters, including salinity, pH, dissolved oxygen, nitrate, and phosphate concentrations. Concurrently, data on stocking density and the presence or absence of cryptocaryoniasis in cultured fish were recorded at each sampling point. These observations were used to benchmark model predictions against empirical disease occurrence, enabling direct accuracy assessment. In parallel, model validation was performed in a controlled RAS environment at the Ningbo University pilot facility located in Meishan, China (Fig. 2). Weekly sampling was carried out to monitor water quality parameters and track disease incidence. Data from both systems were input into the predictive model to generate outbreak forecasts. Comparison between predicted and observed outcomes allowed for quantification of model accuracy under field-relevant aquaculture conditions.

**Table 1:** Data collection on large yellow croaker aquaculture farms

Location (year)	Month	Water temperature (℃)	Salinity (‰)	pH	Dissolved oxygen (mmol/m^3^)	Nitrate (mmol/m^3^)	Phosphate (mmol/m^3^)	Density (kg/m^3^)	Disease outbreak
Shipu (2022)	June	25.30	22.86	8.05	231.46	8.83	0.0029	1.66	Yes
	July	30.51	25.77	7.99	209.81	4.08	0.0009	1.53	Yes
	August	30.61	28.17	8.01	207.21	2.87	0.0012	1.45	Yes
	September	25.64	16.64	8.17	228.83	13.60	0.0020	1.86	No
	October	19.97	18.18	8.20	255.03	11.53	0.0048	1.82	No
	November	19.50	19.21	8.18	258.12	12.28	0.0044	2.18	No
Huangbiao (2022)	June	23.83	29.51	8.00	245.74	7.02	0.0025	2.66	Yes
	July	31.88	19.66	8.05	225.57	15.24	0.0008	2.66	Yes
	August	29.84	21.21	8.08	217.92	14.73	0.0013	5.56	Yes
	September	25.36	16.65	8.22	242.94	30.65	0.0039	8.89	No
	October	19.02	11.53	8.15	270.03	27.23	0.0069	8.89	No
	November	19.36	16.39	8.07	266.35	23.55	0.0087	11.11	No

### Web-based disease prediction platform development

To enhance the usability and interpretability of the ML models, often criticized for their “black box” nature, we developed an interactive web-based platform (https://mapl.shinyapps.io/cryptocaryoniasis_prediction/) for the visualization, deployment, and operationalization of the cryptocaryoniasis prediction framework. The application was built using Shiny, a robust R-based web development environment widely adopted in epidemiological modeling and data-driven disease forecasting [36]. The interface comprises four core modules: (I) data management, (II) model construction, (III) disease prediction, and (IV) spatiotemporal trend mapping.

(I) Data management module. This module supports the import, export, storage, processing, updating, and querying of historical disease outbreak records. Upon dataset updates, the system automatically extracts geolocation and temporal information and retrieves the corresponding oceanographic and meteorological parameters via Copernicus Marine (https://marine.copernicus.eu) and Atmosphere (https://atmosphere.copernicus.eu) Monitoring service APIs. In addition, the platform is scheduled to fetch near-real-time environmental data daily, combining the previous week’s observations with short-term forecast data (up to one week ahead), thereby enabling dynamic and continuous dataset enrichment to support predictive cycles.
(II) Model construction module. This module trains predictive models using five ML algorithms based on the curated datasets. For each algorithm, key performance metrics, including accuracy, precision, recall, specificity, and the confusion matrix, are computed. The platform then automatically ranks the candidate models and selects the optimal one based on overall predictive performance. This selected model is retained for deployment in downstream forecasting tasks.
(III) Disease prediction module. This module allows users to input site-specific geographical coordinates and environmental parameters to generate a risk forecast for cryptocaryoniasis. If direct field measurements are unavailable, the platform can fetch environmental data automatically based on the provided location. The module accommodates both real-time prediction and user-defined simulations, thereby supporting personalized and site-specific risk assessments across different aquaculture operations.
(IV) Spatiotemporal trend mapping module. This module forecasts and visualizes the spatial distribution and weekly outbreak trend of cryptocaryoniasis in coastal Chinese waters. The forecast area spans 105–130° E longitude and 15°–40°N latitude. By leveraging the optimal ML model and integrating near-real-time Copernicus-derived environmental observations together with short-term forecasts (up to one week ahead), the system generates dynamic outbreak risk maps updated daily. Additionally, the platform archives the previous week’s prediction outputs, allowing users to conduct retrospective analysis and track outbreak dynamics over time.

## Results

### Importance of variables

**Fig. 3:**
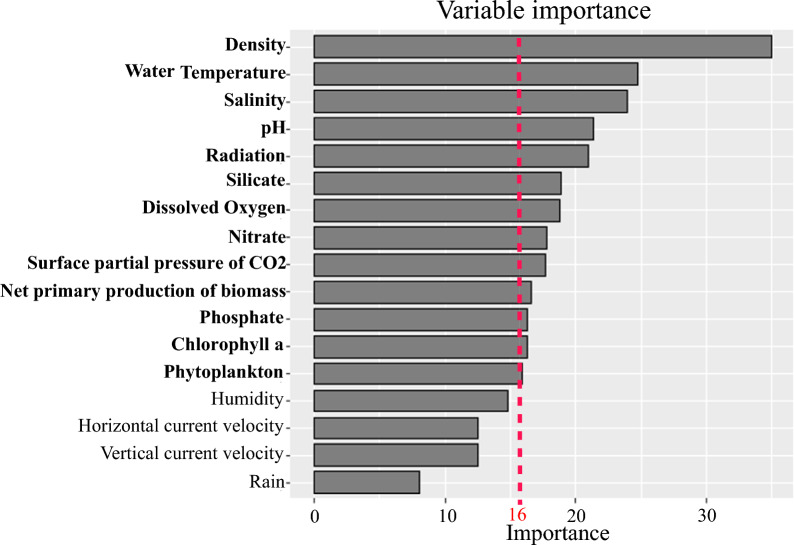
Variable importance ranked by mean decrease in accuracy (MDA) in the random forest model. Features with MDA equal to or higher than 16 were identified as key predictors of cryptocaryoniasis outbreaks, while variables with lower indexes were not included among the top predictors but remained available in model development. Higher MDA values indicate stronger predictive influence

**Fig. 4:**
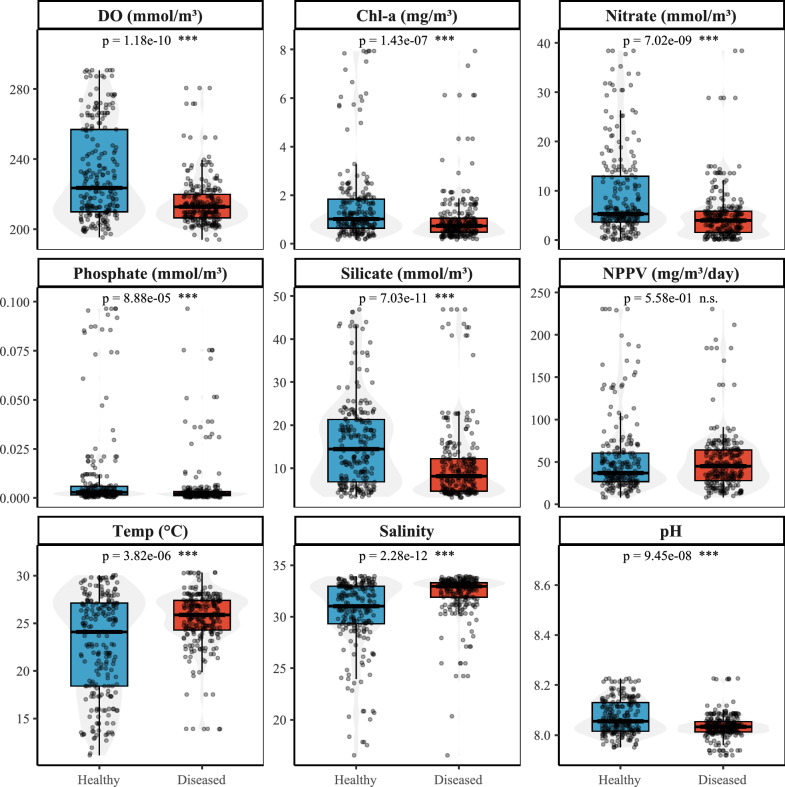
Comparative distributions of key environmental and host-related predictors between outbreak and nonoutbreak cases of cryptocaryoniasis. Boxplots display means and variability (±S.D.), highlighting significant differences (*P* < 0.05) in the nine represented parameters

Feature importance was assessed using the RF algorithm based on the mean decrease in accuracy (MDA). Variables with MDA scores equal to or above 16 were identified as key predictors of cryptocaryoniasis outbreaks and retained for model development. These included stocking density, water temperature, salinity, pH, solar radiation, silicate, dissolved oxygen, nitrate, surface partial pressure of CO_2_, net primary production of biomass, phosphate, and chlorophyll a (Fig. 3). Variables with less MDA from the threshold were considered less influential but remained available within the model framework. Comparative environmental analyses showed that outbreak events were associated with higher seawater temperature and salinity, and lower concentrations of silicates, phosphates, nitrates, dissolved oxygen, chlorophyll, and net primary biomass production (Fig. 4).

### Annual patterns of disease occurrence

**Fig. 5:**
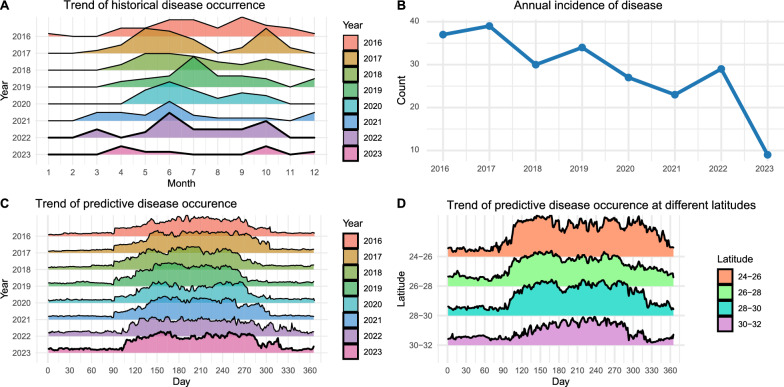
Temporal and spatial patterns in cryptocaryoniasis occurrence. **A** Monthly outbreak frequency from 2016 to 2023. **B** Corresponding model-based outbreak predictions. **C** Disease incidence mapped by latitude along the Chinese coastline. **D** Latitudinal gradient in predicted outbreak probability

Historical records revealed consistent seasonal patterns in outbreak incidence. Two peaks were observed annually: a primary peak in May–July and a secondary peak in September–November. Outbreak occurrence was lowest during January–February and August (Fig. 5A). These seasonal trends were highly reproducible across years, highlighting the role of environmental and climatic conditions in outbreak dynamics. A general decline in outbreak frequency was noted from 2016 to 2023 (Fig. 5B).

### Performance evaluation of machine learning models

After feature selection, all five ML classifiers (LR, SVM, RF, XGB, and ANN) were trained and tested. Performance metrics are summarized in Table 2. XGB achieved the highest overall accuracy (92.97%), while RF showed the highest sensitivity (98.61%), closely followed by XGB (95.83%). The F1 score also confirmed XGB as the top-performing model (93.88%).

**Table 2:** Evaluation of disease prediction performance on test set

Classfier^a^	Accuracy	Sensitivity	Specificity	Precision	F1-score
LR	0.7422	0.7361	0.7500	0.7910	0.7626
SVM	0.8359	0.8333	0.8393	0.8696	0.8511
RF	0.9141	0.9861	0.8214	0.8765	0.9281
XGB	0.9297	0.9583	0.8929	0.9200	0.9388
ANN	0.8359	0.8750	0.7857	0.8400	0.8571

^a^*LR* liner regression, *SVM* support vector machines, *RF* random forest, *XGB* extreme gradient boosting, *ANN* artificial neural network

**Fig. 6:**
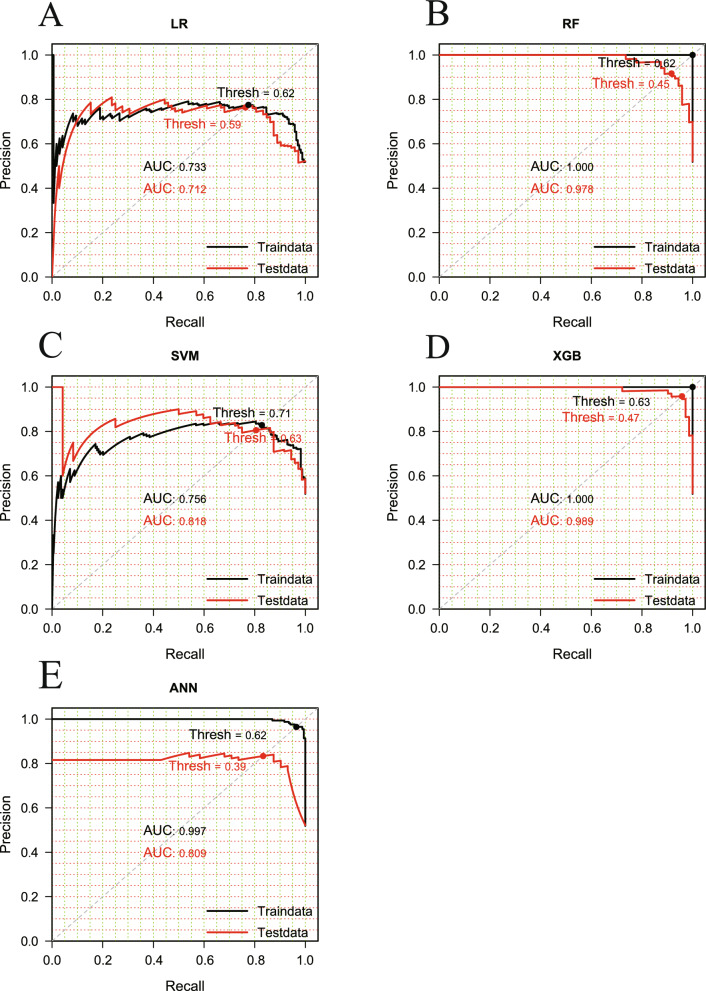
Precision-recall curve evaluating model performance. Graphs show results for **A** logistic regression (LR), **B** random forest (RF), **C** support vector machine (SVM), **D** XGBoost (XGB), and **E** artificial neural network (ANN) models, based on test set predictions

Precision-recall (PR) curves and area under the curve (AUC) values for both training and testing datasets further validated model performance (Fig. 6). RF and XGB models achieved perfect AUCs (1.0) on the training data and sustained high predictive accuracy on the testing set, with AUCs of 0.978 and 0.989, respectively. In contrast, the baseline LR model observed the weakest performance, with an AUC of 0.733. These results highlight the superior predictive power and generalization capacity of the ensemble models. Model-based forecasts also mirrored historical outbreak patterns, capturing both seasonal peaks and latitudinal gradients (Fig. 5C, D). Outbreaks were most frequent and variable at low-latitude zones (24–26°N), with incidence rates and variability declining progressively northward (Fig. 5D). We recognize, however, that in 2023 the observed occurrence of cryptocaryoniasis was low at several monitoring sites (Fig. 5A, B), while the model predicted relatively high risk (Fig. 5C). This apparent discrepancy reflects the difference between regional-scale predictions, which represent agreed coastal trends, and localized field records, which are inherently limited to specific sites. To clarify this point, we have included a Supplementary figure (Fig. S1) that directly compares model predictions with observed disease records at the four monitoring sites in 2023. In this figure, black dots denote observed disease, red dots denote absence of disease, and the prediction curve represents model probabilities of occurrence. Probabilities above 05 indicate higher outbreak risk, while values below 0.5 indicate low risk. Across all four sites, the model achieved >80% prediction accuracy, reinforcing its robustness and emphasizing the importance of scale when interpreting model outputs.

### Field verification of model performance in open-sea cages

Model validation using field data from open-sea cage systems is presented in Table 3. The RF and SVM models achieved the highest accuracy (91.67%), with both demonstrating strong predictive performance, including a sensitivity of 83.33% and perfect specificity. The XGB model demonstrated the highest sensitivity (100%), though its accuracy (75%) matched the baseline LR model (75%), with a lower F1 score (0.7692) owing to a higher false positive rate. The ANN model achieved an accuracy of 83.33% with balanced sensitivity and specificity. Overall, the RF and SVM models showed robust performance, while XGB’s high sensitivity highlighted its effectiveness in detecting true outbreaks.

**Table 3:** Evaluation of model performance using data collected from large yellow croaker farms in open-sea cages

Classfier^a^	Accuracy	Sensitivity	Specificity	Precision	F1-score
LR	0.7500	0.5000	1.0000	1.0000	0.6667
SVM	0.9167	0.8333	1.0000	1.0000	0.9091
RF	0.9167	0.8333	1.0000	1.0000	0.9091
XGB	0.7500	1.0000	0.6667	0.7143	0.7692
ANN	0.8333	0.8333	0.6667	0.7500	0.8571

^a^*LR* liner regression, *SVM* support vector machines, *RF* random forest, *XGB* extreme gradient boosting, *ANN* artificial neural network

### RAS trial validation

In RAS, the XGB model achieved an accuracy of 87.5% and the highest F1 score of 0.923 (Table 4), demonstrating robust diagnostic performance with a sensitivity of 88.9% and a precision of 96.0%, indicating a well-balanced capability for reliable detection of cryptocaryoniasis. The LR model outperformed with an accuracy of 96.88%, while the RF model achieved 93.75% accuracy, both showing strong specificity (100 and 96.3%, respectively). The SVM model performed the weakest with an accuracy of 38.24%. Although the ANN model achieved 100% sensitivity by correctly identifying all positive cases, its higher false positive rate resulted in lower accuracy (56.25%) and reliability. Additionally, outbreak events in the RAS were confirmed by trained aquaculture personnel on the basis of standardized diagnostic procedures. Initial identification relied on gross clinical signs, particularly the presence of characteristic nodules on the gills, skin, and fins. This was followed by wet-mount microscopic examination for direct visualization of *C. irritans* trophonts. Molecular identification was not required in any case.

**Table 4:** Evaluation of model performance using data collected from RAS

Classfier^a^	Accuracy	Sensitivity	Specificity	Precision	F1-score
LR	0.9688	0.8000	1.0000	1.0000	0.8889
SVM	0.3824	0.2857	0.4074	0.1111	0.1600
RF	0.9375	0.8000	0.9630	0.8000	0.8000
XGB	0.8750	0.8890	0.8000	0.9600	0.9230
ANN	0.5625	1.0000	0.4815	0.2632	0.4167

^a^*LR* liner regression, *SVM* support vector machines, *RF* random forest, *XGB* extreme gradient boosting, *ANN* artificial neural network

### Web-based disease prediction platform x

**Fig. 7:**
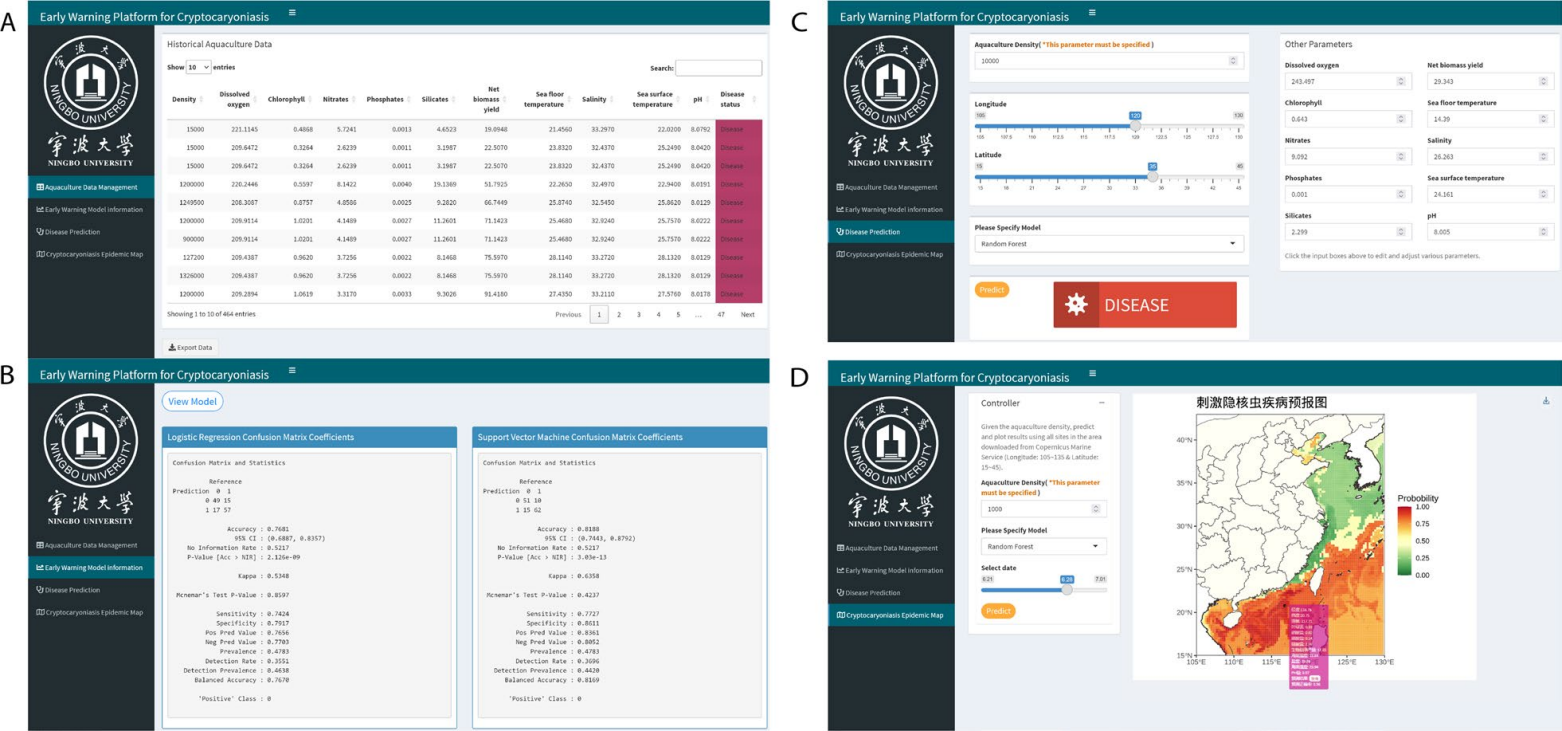
Screenshots of the web-based early warning platform for cryptocaryoniasis risk prediction. The interface allows users to **A** input site-specific parameters, **B** visualize predicted outbreak probabilities, **C** access temporal trend forecasts, and **D** explore spatial risk distribution along the coastline. Note: The four screenshots represent the main functions available in the software menu, included here to provide readers with a complete overview of the web-based platform. Detailed interaction with these functions can be explored directly in the web application. The platform is accessible at https://mapl.shinyapps.io/cryptocaryoniasis_prediction/ for real-time interaction

To enhance accessibility, interpretability, and user engagement, we developed an interactive web-based early warning platform for cryptocaryoniasis. The site is publicly available at the following link: https://mapl.shinyapps.io/cryptocaryoniasis_prediction/. The platform consists of four integrated modules, each hosted on a dedicated page:

*Data Management Page*: Presents historical outbreak records in tabular format with search, sort, download, and update functions. It also integrates Copernicus-derived environmental data via API integration, enriching the dataset automatically (Fig. 7A).

*Model Information Page*: provides a summary of model evaluation metrics, including confusion matrices, accuracy, precision, sensitivity, specificity, and F1 scores, for all five machine learning models (Fig. 7B).

*Disease Prediction Page*: Allows users to input farm coordinates and stocking density to generate real-time risk predictions. Environmental data can be auto-fetched or manually entered (Fig. 7C).

*Epidemic Map Page*: Displays weekly outbreak probabilities along China’s coastline (105–130° E, 15–40° N). A color-coded scheme indicates risk levels from high (red) to low (green), with hover functionality offering localized environmental data for informed decision-making (Fig. 7D).

## Discussion

Aquatic diseases continue to pose a significant threat to global aquaculture, leading to major economic losses and compromising food quality and security [37]. Timely forecasting is essential for effective disease mitigation. This study demonstrates that machine learning (ML) models offer a robust and accurate tool for early detection of cryptocaryoniasis, one of the most damaging parasitic diseases in marine aquaculture. The optimized prediction model achieved high overall accuracy (91.67%), precision (87–89%), and recall (89%), highlighting its reliability under operational conditions.

Random forest-based feature importance analysis identified stocking density, water temperature, salinity, and pH as the most influential predictors of disease outbreaks. These findings align with previous studies showing that rearing density strongly modulates *C. irritans* transmission dynamics in *L. crocea* [5, 38]. Notably, our findings indicate that while moderate host densities can exacerbate infection by increasing parasite–host encounters and accelerating outbreak speed, very high densities may distribute the parasite load across many individuals, reducing the per-fish burden and thereby allowing partial immune control that slows disease progression [39]. In addition, a supplementary comparison of model predictions and observed records at the four monitoring sites in 2023 (Fig. S1) further illustrates that the model maintained >80% accuracy, while highlighting the importance of scale when interpreting regional forecasts against local field data. Water temperature, a well-known driver of *C. irritans* proliferation, modulates both infection window (18–30 °C) and life cycle duration (7–14 days), with peak virulence at 24–27 °C [8, 40]. Similarly, deviations in salinity and pH also affect tomont encystation and theront viability, thereby influencing the reproductive potential of *C. irritans* [10, 41].

Our findings further emphasize the importance of solar radiation and dissolved oxygen, previously suggested as modulators of outbreak occurrence. In fact, *C. irritans* can enter a dormant state under hypoxic conditions and excyst in response to oxygen repletion and photoperiod cues, with emerging evidence suggesting that light-induced hatching may initiate cryptocaryoniasis outbreaks [42–46].

In addition to known factors, our model highlighted potential contributors of underexplored variables such as silicate, nitrate, and phosphate. These nutrients may influence seawater calcium and magnesium ion availability, which are believed to be key components in tomont cyst wall formation of *C. irritans* [47]. Silicic acid and phosphoric acids can precipitate calcium and magnesium ions, reducing their availability in the water column. Therefore, the lower concentrations of these ions may impair encystment, potentially decreasing infection probability [10, 48]. These associations merit further investigation to clarify the mechanisms linking nutrient dynamics and parasite development.

Advanced computational approaches are increasingly applied to disease forecasting, but compared with other parasitic disease models, our RF-based approach stands out due to its integration of real-time environmental data via Copernicus services, offering dynamic risk assessments. For instance, logistic regression and neural networks have been used to predict shrimp diseases based on farm location and management practices [49], support vector machines have been used to estimate freshwater disease risk in the UK [50], and multivariate regression models have been developed to forecast diseases in large yellow croaker based on environmental data in China [51, 52]. However, these are typically limited by reliance on static water quality datasets and lack the ability to forecast future outbreaks, highlighting the advantage of our approach’s real-time capabilities.

While real-time water quality monitoring sensors are ideal, many small-scale farms, particularly in developing countries, cannot afford such technologies. Moreover, low-cost sensors can only monitor a limited set of variables, while more informative environmental indicators (e.g., solar radiation, chlorophyll, nutrients) require satellite or ocean buoys data [53, 54]. Previous models have also lacked user-friendly interfaces, limiting their practical application in farm operations.

In contrast, large-scale marine monitoring initiatives, such as Copernicus Marine Services, provide freely accessible, high-resolution forecasts of oceanographic and atmospheric variables through open APIs [55, 56]. By integrating this data, our model allows users to predict disease risk without on-site measurements or expensive equipment. This significantly lowers the barrier to implementation, enabling widespread adoption, especially in resource-limited settings.

Using Copernicus data, we developed a web-based early warning system for predicting cryptocaryoniasis risk along China’s coastline. The platform supports real-time and weekly forecasts and includes tools for data updating, model retraining, and visualization. It provides actionable information for frontline aquaculture workers and allows continuous improvement as more outbreak data becomes available. Furthermore, we assessed the performance of the disease prediction model in both a closed RAS and an open-sea cage environment. The results indicate that the disease prediction model maintains a satisfactory level of accuracy in both environments.

Despite these advances, some limitations remain; disease emergence is influenced by interactions among pathogens, hosts, and environmental variables. While environmental data can be continuously monitored and modeled, host and pathogen dynamics still rely heavily on molecular tools with superior sensitivity, tools that are not yet fully integrated into automated systems. Our prior research has investigated how environmental variables influence both parasite life cycles and host immune responses in laboratory settings and has employed image recognition to detect *C. irritans* infections on the basis of characteristic white spots on infected fish [10, 39, 41].

Future model improvements should incorporate additional data on pathogen prevalence and host immune status. Past outbreaks can also influence future disease events, as dormant tomonts may settle and reactivate under favorable conditions. Treatment practices, ocean currents, and fish migration can all modulate infection spread and recurrence. Incorporating these dynamics will require a more comprehensive, systems-level approach.

Finally, while our platform was designed for cryptocaryoniasis, its architecture is adaptable. It could be extended to forecast other parasitic diseases, such *as Ichthyophthirius multifiliis* and *Ichthyobodo* spp. By integrating multidisciplinary datasets and accounting for the complex life cycles of aquatic pathogens, this model lays the foundation for a universal early warning system applicable across diverse aquatic environments.

## Conclusions

This study demonstrates the effectiveness of machine learning in predicting cryptocaryoniasis outbreaks in marine aquaculture. By integrating historical outbreak records with high-resolution marine biogeochemical data, we developed a predictive model capable of delivering accurate, real-time assessments. Among the models tested, the RF algorithm emerged as the most effective, achieving an F1 score of 0.9388, a sensitivity of 98.61%, and a precision of 87.65%, with its superior sensitivity and balanced accuracy across field and RAS settings making it the preferred choice over XGB. This latest model, despite achieving higher overall accuracy (92.97%) and an F1 score of 93.88, showed lower performance in the open sea. Field validation confirmed the RF model accuracy, with only a slight misclassification among all predictions conducted in open-sea conditions. Temperature was identified as the most critical risk factor, consistent with prior studies. The inclusion of additional variables such as salinity, pH, dissolved oxygen, and solar radiation further improved predictive performance. To support practical application, we developed a user-friendly, web-based early warning platform. This system enables aquaculture operators to access weekly outbreak forecasts, visualize spatiotemporal risk trends, and make informed management decisions. The platform’s ability to integrate real-time environmental data, without requiring on-site sensors, makes it particularly suitable for small-scale farms and resource-limited settings. Overall, the model provides a robust, scalable framework for disease surveillance in marine aquaculture. Beyond cryptocaryoniasis, it offers a flexible foundation for developing early warning systems targeting a broader range of aquatic pathogens, advancing precision health management across diverse global aquaculture settings. Further iterations could integrate IoT sensors with real-time host–pathogen data to enhance precision.

## Supplementary Information

Additional file 1: Supplemental Figure 1. Comparison of model-predicted outbreak probabilities and observed cryptocaryoniasis records at four monitoring sites in 2023. The solid line represents the predicted probability of outbreak occurrence over time. Black dots indicate field records of disease occurrence, and red dots indicate the absence of disease. Predicted probabilities above 0.5 denote high outbreak risk, while values below 0.5 denote low risk. Across all sites, prediction accuracy exceeded 80%, supporting the robustness of the model while highlighting differences between regional-scale forecasts and local observations.

## Data Availability

Data supporting the main conclusions of this study are included in the manuscript. Analysis code is publicly available at: https://gitee.com/Chauncy27/machine-learning-based-disease-predictive-platform-for-marine-fish-cryptocaryoniasis/.

## Abbreviations

ANN: Artificial neural network
API: Application programming interface
AUC: Area under the curve
AUROC: Area under the receiver operating characteristic
E: East
FN: False negatives
FP: False positives
LR: Logistic regression
MDA: Mean decrease in accuracy
ML: Machine learning
N: North
OOB: Out-of-bag
PR: Precision-recall
RASS: Recirculating aquaculture systems
RF: Random forest
RFE: Recursive feature elimination
SVM: Support vector machine
TP: True positives
XGB: XGBoost

## References

[1] FAO. The state of world fisheries and aquaculture. Rome: FAO; 2024.

[2] Rowley AF, Baker-Austin C, Boerlage AS, Caillon C, Davies CE, Duperret L, et al. Diseases of marine fish and shellfish in an age of rapid climate change. iScience. 2024;27:110838. 10.1016/j.isci.2024.110838. 39318536 10.1016/j.isci.2024.110838 PMC11420459

[3] Chi H, Taik P, Foley EJ, Racicot AC, Gray HM, Guzzetta KE, et al. High genetic diversities between isolates of the fish parasite *Cryptocaryon irritans* (Ciliophora) suggest multiple cryptic species. Mol Phylogenet Evol. 2017;112:47–52. 10.1016/j.ympev.2017.04.015. 28428147 10.1016/j.ympev.2017.04.015

[4] Xie X, Zheng C, Zahid A, Kong J, Bushra, Qian D, et al. Updating specific PCR primer for detection of *Cryptocaryon irritans* from reared *Larimichthys polyactis*. Exp Parasitol. 2021;223:108081. 33549536 10.1016/j.exppara.2021.108081

[5] Li Y, Jiang B, Mo Z, Li A, Dan X. *Cryptocaryon irritans* (Brown, 1951) is a serious threat to aquaculture of marine fish. Rev Aquacult. 2022;14:218–36.

[6] Guo X, Huang W, Xu Y, Zhan Q, Sun P, Hu H. Metabolomic changes in *Cryptocaryon irritans* from *Larimichthys crocea* after exposure to copper plate. Front Cell Infect Microbiol. 2024;14:1424669. 39006747 10.3389/fcimb.2024.1424669 PMC11239337

[7] Galindo-Villegas J, Bossier P, Reyes-López FE. Editorial: Oral immune-enhancing research in fish. Front Immunol. 2022;13:850026. 35371028 10.3389/fimmu.2022.850026 PMC8967974

[8] Yin F, Yin J, Xie X, Jiang L. Water temperature affects *Cryptocaryon irritans* development, cryptocaryoniasis occurrence and an auxiliary treatment decision-making webpage. Aquaculture. 2023;574:739694.

[9] Standing D, Brunner T, Aruety T, Ronen Z, Gross A, Zilberg D. Mortality of *Cryptocaryon irritans* in sludge from a digester of a marine recirculating aquaculture system. Aquaculture. 2017;467:134–7.

[10] Zhou L, Huang J, Jiang Y, Kong J, Xie X, Yin F. pH regulates the formation and hatching of *Cryptocaryon irritans* tomonts, which affects cryptocaryoniasis occurrence in *Larimichthys crocea* aquaculture. Appl Environ Microbiol. 2022;88:e0005822. 35254098 10.1128/aem.00058-22 PMC9004364

[11] Selbach C, Mouritsen KN, Poulin R, Sures B, Smit NJ. Bridging the gap: aquatic parasites in the one health concept. Trends Parasitol. 2022;38:109–11. 34863638 10.1016/j.pt.2021.10.007

[12] Brugere C, Kumar G, Bondad-Reantaso MG. Bridging aquatic organism health and economics in the analysis of disease impacts and biosecurity strategies in aquaculture: a conceptual framework. Crit. Insights Aquac. 2025;1,2441506. 10.1080/29932181.2024.2441506.

[13] Gozlan RE, Bommarito C, Caballero-Huertas M, Givens J, Mortillaro J-M, Pepey E, et al. A one-health approach to non-native species, aquaculture, and food security. Water Biol Security. 2024;3:100250.

[14] Zhao S, Zhang S, Liu J, Wang H, Zhu J, Li D, et al. Application of machine learning in intelligent fish aquaculture: a review. Aquaculture. 2021;540:736724.

[15] Tonnang HE, Salifu D, Mudereri BT, Tanui J, Espira A, Dubois T, et al. Advances in data-collection tools and analytics for crop pest and disease management. Curr Opin Insect Sci. 2022;54:100964. 36055644 10.1016/j.cois.2022.100964

[16] Dahle SW, Gaarden SI, Buhaug JF, Netzer R, Attramadal KJK, Busche T, et al. Long-term microbial community structures and dynamics in a commercial RAS during seven production batches of Atlantic salmon fry (*Salmo salar*). Aquaculture. 2023;565:739155.

[17] von Schuckmann K, Le Traon P-Y, Smith N, Pascual A, Brasseur P, Fennel K, et al. Copernicus marine service ocean state report. J Oper Oceanogr. 2018;11:S1–142.

[18] Hijmans RJ. terra: Spatial Data Analysis. The R Foundation. 2024.

[19] R Core T. R: A Language and Environment for Statistical Computing. Computer software. Vienna, Austria.: Foundation for Statistical Computing; 2024.

[20] Breiman L. Random forests. Mach Learn. 2001;45:5–32.

[21] Little R, Rubin D. Statistical analysis with missing data. 3rd ed. New York: Wiley; 2019.

[22] Lunardon N, Menardi G, Torelli N. ROSE: a package for binary imbalanced learning. R J. 2014;6:79.

[23] Chawla NV, Bowyer KW, Hall LO, Kegelmeyer WP. SMOTE: synthetic minority over-sampling technique. JAIR. 2002;16:321–57.

[24] Wickham H, François R, Henry L, Müller K, Vaughan D. dplyr: A Grammar of Data Manipulation. The R Foundation. 2024.

[25] Wickham H, Vaughan D, Girlich M. tidyr: Tidy Messy Data. The R Foundation. 2024.

[26] Gregorutti B, Michel B, Saint-Pierre P. Correlation and variable importance in random forests. Stat Comput. 2017;27:659–78.

[27] Meyer D, Dimitriadou E, Hornik K, Weingessel A, Leisch F. e1071: Misc Functions of the Department of Statistics, Probability Theory Group (Formerly: E1071), TU Wien. The R Foundation. 2024.

[28] Breiman L, Cutler A, Liaw A, Wiener M. randomForest: Breiman and Cutlers Random Forests for Classification and Regression. The R Foundation. 2024.

[29] Chen T, He T, Benesty M, Khotilovich V, Tang Y, Cho H, et al. xgboost: extreme gradient boosting. Vienna: The R Foundation; 2024.

[30] Günther F, Fritsch S. Neuralnet: training of neural networks. R J. 2010;2:30.

[31] Bai Q, Su C, Tang W, Li Y. Machine learning to predict end stage kidney disease in chronic kidney disease. Sci Rep. 2022;12:8377. 35589908 10.1038/s41598-022-12316-z PMC9120106

[32] Niimi N, Shiraishi Y, Sawano M, Ikemura N, Inohara T, Ueda I, et al. Machine learning models for prediction of adverse events after percutaneous coronary intervention. Sci Rep. 2022;12:6262. 35428765 10.1038/s41598-022-10346-1 PMC9012739

[33] Xu X, Fairley CK, Chow EPF, Lee D, Aung ET, Zhang L, et al. Using machine learning approaches to predict timely clinic attendance and the uptake of HIV/STI testing post clinic reminder messages. Sci Rep. 2022;12:8757. 35610227 10.1038/s41598-022-12033-7 PMC9128330

[34] Han D-H, Lee S, Seo D-C. Using machine learning to predict opioid misuse among US adolescents. Prev Med. 2020;130:105886. 31705938 10.1016/j.ypmed.2019.105886

[35] Haq AU, Li JP, Khan J, Memon MH, Nazir S, Ahmad S, et al. Intelligent machine learning approach for effective recognition of diabetes in E-healthcare using clinical data. Sensors 2020;20, 2649. 10.3390/s20092649. 32384737 10.3390/s20092649 PMC7249007

[36] Jia L, Yao W, Jiang Y, Li Y, Wang Z, Li H, et al. Development of interactive biological web applications with R/Shiny. Brief Bioinform. 2022;23:bbab415. 10.1093/bib/bbab415. 34642739 10.1093/bib/bbab415

[37] Zhou R, Sun K, Xie X, Yin F and Galindo-Villegas J, et al. Integrated transcriptomic and immune enzymatic analyses uncover coordinated immunometabolic responses in large yellow croaker (Larimichthys crocea) to Metanophrys sp. infection. Front. Immunol. 2025;16:1636453. 10.3389/fimmu.2025.1636453 10.3389/fimmu.2025.1636453 PMC12307142 40740775

[38] Yanong RP. *Cryptocaryon irritans* infections (marine white spot disease) in fish. EDIS. 2010. 10.32473/edis-fa164-2009.

[39] Zhou L, Zhou R, Xie X, Yin F. Characteristics and risk assessment of cryptocaryoniasis in large yellow croaker (*Larimichthys crocea*) at different densities in industrialized aquaculture. Aquaculture. 2024;582:740501.

[40] Wang J-L, Lao G-F, Li Y-W, Yang M, Mo Z-Q, Dan X-M. Effects of temperature and host species on the life cycle of *Cryptocaryon irritans*. Aquaculture. 2018;485:49–52.

[41] Kong J, Zhou L, Yuan Y, Wang L, Kang T, Wu J, et al. Salinity regulates the formation and hatching of *Cryptocaryon irritans* tomonts, affecting infectivity to *Larimichthys crocea*. Aquaculture. 2022;554:738166. 10.1128/aem.00058-22 PMC9004364 35254098

[42] Burgess PJ, Matthews RA. *Cryptocaryon irritans* (Ciliophora): photoperiod and transmission in marine fish. J Mar Biol Ass. 1994;74:535–42.

[43] Kotake M, Watanabe Y, Itoh N, Yoshinaga T. Effect of light exposure on circadian rhythm in theront excystment in *Cryptocaryon irritans*. Parasitol Int. 2024;98:102812. 37777053 10.1016/j.parint.2023.102812

[44] Skilton DC, Saunders RJ, Hutson KS. Parasite attractants: identifying trap baits for parasite management in aquaculture. Aquaculture. 2020;516:734557.

[45] Watanabe Y, How KH, Zenke K, Itoh N, Yoshinaga T. Dormancy induced by a hypoxic environment in tomonts of *Cryptocaryon irritans*, a parasitic ciliate of marine teleosts. Aquaculture. 2018;485:131–9.

[46] Watanabe Y, How KH, Zenke K, Itoh N, Yoshinaga T. Control of the daily rhythms by photoperiods in protomont detachment and theront excystment of the parasitic ciliate *Cryptocaryon irritans*. Fish Pathol. 2020;55:38–41.

[47] Ma R, Fan X, Yin F, Ni B, Gu F. Ultrastructural features of the tomont of *Cryptocaryon irritans* (Ciliophora: Prostomatea), a parasitic ciliate of marine fishes. Parasitology. 2017;144:720–9. 28134067 10.1017/S0031182016002651

[48] Wang X, Xie W, Li T, Ren J, Zhu J, Han N, et al. Molecular dynamics study on mechanical properties of interface between urea-formaldehyde resin and calcium-silicate-hydrates. Materials 2020;13, 4054. 10.3390/ma13184054. 32932664 10.3390/ma13184054 PMC7558882

[49] Leung P, Tran LT. Predicting shrimp disease occurrence: artificial neural networks vs. logistic regression. Aquaculture. 2000;187:35–49.

[50] Hassani H, Silva E, Combe M, Andreou D, Ghodsi M, Yeganegi M, et al. A support vector machine based approach for predicting the risk of freshwater disease emergence in England. Stats. 2019;2:89–103.

[51] Cai XP. Preliminary establishment of large yellow croak disease diagnosis and pre-warning system. Doctoral dissertation. Xiamen University; 2013.

[52] Zhou RJ. Studies on the forecasting of the main disease in cage-cultured Pseudosciaena crocea. Doctoral dissertation. Ningbo University; 2011.

[53] Gasparin F, Guinehut S, Mao C, Mirouze I, Rémy E, King RR, et al. Requirements for an integrated in situ Atlantic Ocean observing system from coordinated observing system simulation experiments. Front. Mar. Sci. 2019;6, 83. 10.3389/fmars.2019.00083.

[54] Mohd Jais NA, Abdullah AF, Mohd Kassim MS, Abd Karim MM, Abdulsalam M, Muhadi NA. Improved accuracy in IoT-Based water quality monitoring for aquaculture tanks using low-cost sensors: Asian seabass fish farming. Heliyon. 2024;10:e29022. 38655304 10.1016/j.heliyon.2024.e29022 PMC11035052

[55] Danovaro R, Carugati L, Berzano M, Cahill A, Carvalho S, Chenuil A, et al. Implementing and innovating marine monitoring approaches for assessing marine environmental status. Front. Mar. Sci. 2016;3, 213. 10.3389/fmars.2016.00213.

[56] Ma J, Ma R, Pan Q, Liang X, Wang J, Ni X. A global review of progress in remote sensing and monitoring of marine pollution. Water. 2023;15:3491.

