# Supplementary figures and images for: A machine learning-driven early warning system for cryptocaryoniasis in marine aquaculture
Source: Parasit Vectors. 2025 Nov 26;18:490. doi: 10.1186/s13071-025-07124-z (PMC12659040; doi:10.1186/s13071-025-07124-z)

A

Sampling Point 1

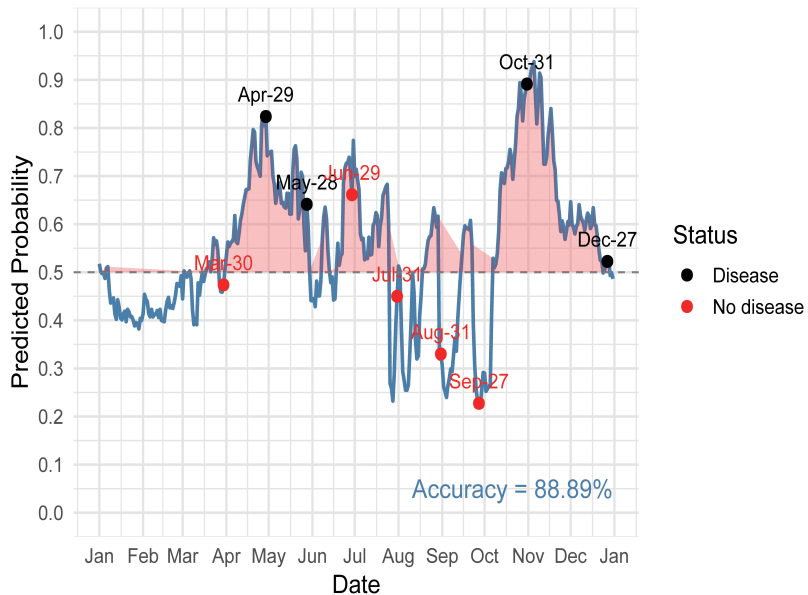

B

Sampling Point 2

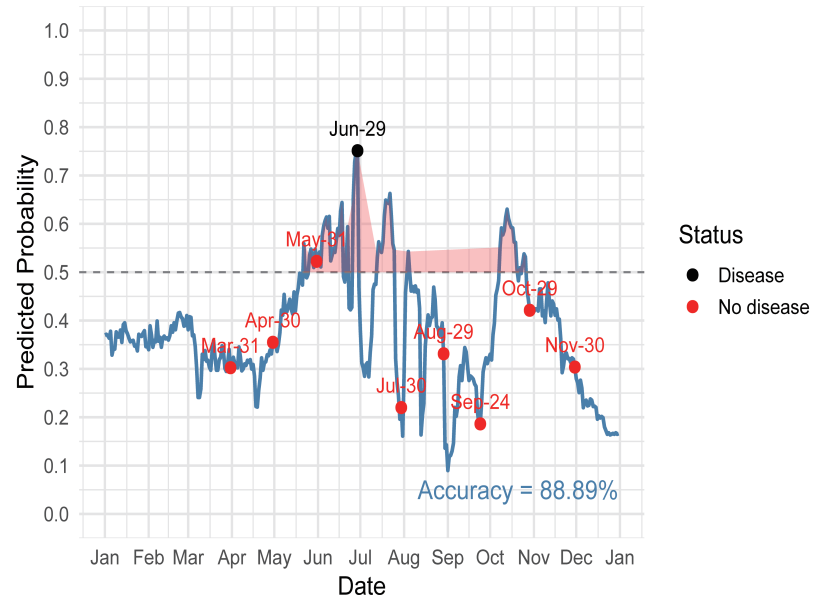

C

Sampling Point 3

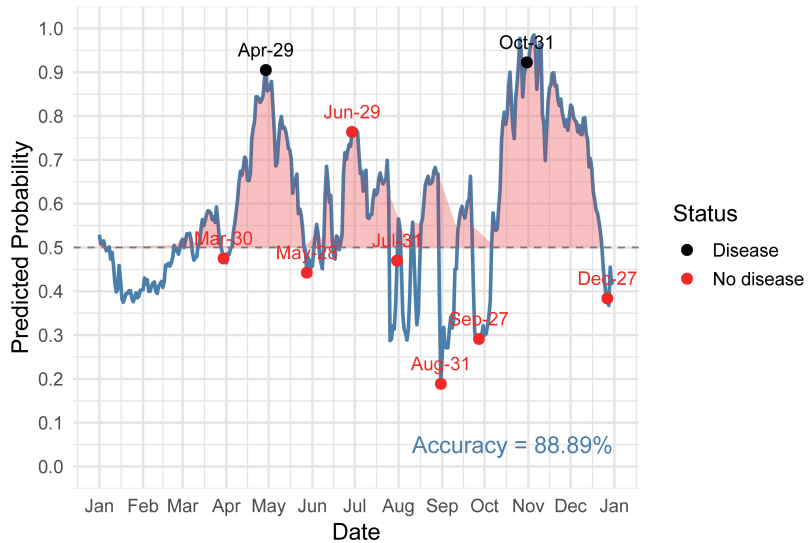

D

Sampling Point 4

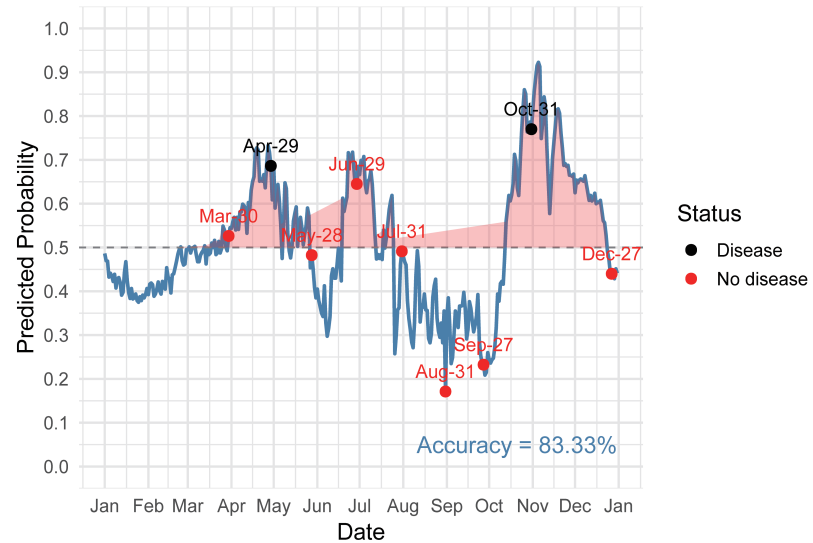

Supplement: Supplementary file 1 — Additional file 1: Supplemental Figure 1. Comparison of model-predicted outbreak probabilities and observed cryptocaryoniasis records at four monitoring sites in 2023. The solid line represents the predicted probability of outbreak occurrence over time. Black dots indicate field records of disease occurrence, and red dots indicate the absence of disease. Predicted probabilities above 0.5 denote high outbreak risk, while values below 0.5 denote low risk. Across all sites, prediction accuracy exceeded 80%, supporting the robustness of the model while highlighting differences between regional-scale forecasts and local observations. [file 13071_2025_7124_MOESM1_ESM.pdf]
